# On Gatekeepers and Structural Competition Problems

**DOI:** 10.1007/s10272-021-0986-5

**Published:** 2021-08-05

**Authors:** Christian Rusche, Jan Büchel

**Affiliations:** 1 Research Unit Digitalization, Structural Change and Competition, German Economic Institute, Konrad-Adenauer-Ufer 21, 50668 Cologne, Germany; 2 P.O. Box 101942, 50459 Cologne, Germany

## Abstract

The Digital Age saw the rise of several rapidly growing digital platforms with substantial market shares, and this development is expected to continue. Europe is a large target market for these globally operating platforms, although the majority of the most successful platforms come from the USA and Asia and will likely continue to do so in the future. This article reveals the reasons for the success of digital platforms and discusses the recent European Commission proposal for a Digital Markets Act based on the analysis of the status quo.

On 15 December 2020, the European Commission published its proposals on the Digital Markets Act (European Commission, 2020b) and the Digital Services Act (European Commission, 2020a). These proposals stand for the latest EU-level effort to adapt the regulatory system to the increased economic power and societal influence of digital platforms. The Digital Markets Act targets a limited number of core platform services of very large online platforms. The Digital Services Act aims to create a transparent and safe online environment. Concentrating on the proposed Digital Markets Act, this article discusses the importance of digital platforms in the European Union and suggests the role they will play in the future. This includes an analysis of the factors that make platforms successful and that allow them to grow at such a rapid speed and discusses the relationship of platforms and competition based on the results of this analysis. Against this backdrop, some platforms are ascribed to be gatekeepers in some markets. It also shows what makes a platform a gatekeeper and under which circumstances a gatekeeper position can cause structural competition problems that justify a market intervention. This also allows for the derivation of policy recommendations that enrich the discussion on the recent regulatory proposals.

* This article is an updated version of Büchel and Rusche (2020a).

## Competition and digital platforms

In the economic literature on competition, there is an ongoing discussion about whether competition is an aim by itself or just a means to reach other goals (Schmidt, 1999, 32). Generally, functioning competition can be a means that helps to achieve a performance-based income distribution, consumer sovereignty, an ideal allocation of production factors, flexibility and technological progress. Despite its beneficial role for an economy, full-scale competition, defined as atomistic players without impact on the market outcome acting on a given market, is not always an ideal choice (Schmidt, 1999). Among others, this holds for digital platforms. A digital platform can be defined as “an enterprise that uses the internet to facilitate economically beneficial interactions between two or more independent groups” (Demary and Rusche, 2018, 8).

There are three success factors for a digital platform (Demary and Rusche, 2018).

*Economies of scale*: In the digital economy, there are no or only a few physical barriers that prevent a scaling up of the business model. The marginal costs for including an additional user or for selling an additional service are, therefore, close to zero. But the starting costs for the business, which are based on the hardware, the software and the marketing costs for attracting users, can be very high.

*Positive indirect network effects*: The platform facilitates transactions between different groups of users. To this end, users from at least one user group must wish to be matched with users from another group. The platform's business model is to decrease the transaction costs for such a match. Furthermore, when deciding on a platform, a user takes the number of potential transaction partners into account. The platform with the largest number is usually most attractive to him. The increased attractiveness of a platform with the number of users in another group is described by positive indirect network effects. Positive indirect network effects on more than one side that reinforce each other allow the platform to grow even faster and gain market power quickly.

*Data analysis*: The platform can collect data from the matching process and the different user groups. The integration of this data from different contexts can be used to extract information that can be especially valuable (Krotova et al., 2019). This information allows, for example, an improvement of the matching process, the revelation of opportunities for new products, services or selling advertising space.

All three success factors highlight that a big platform can yield a better outcome for itself but also for the whole economy. Such a platform will lead to higher household incomes and a better consumer satisfaction than several small platforms. One or a few platforms can be focal points for users to carry out transactions and, therefore, work as a co-ordination device. One large platform reduces transaction costs for all sides of the market. Furthermore, before a dominant platform evolves, there is a fierce competition for the market. Especially self-reinforcing positive indirect network effects can start a stable growth process that allows a platform to quickly dominate a market once a critical mass of users is reached (Evans and Schmalensee, 2016, 78). This relationship is highlighted by the term winner-takes-all market (Demary and Rusche, 2018).

However, if just one platform dominates the market and managed to obtain a durable and stable position in the market, that platform can be termed a gatekeeper. It is vital for users to be active on that platform in order to get in contact with potential transaction partners. The COVID-19 pandemic also facilitated the role of platforms due to lockdowns in several countries. Even in the accommodation sector, which was affected by lockdowns, platforms like Airbnb or Booking.com can benefit in the end. The asset-light business model of platforms – the goods and services offered on the platform are supplied by the users – allows the platform to keep costs low and adapt quickly to changes in demand or supply. Accordingly, the platforms in the accommodation sector suffer smaller damages and can easily benefit from the opportunities once the pandemic is over.

## Platform's role in the economy

The importance of platforms is shown by the fact that eight of the 10 most valuable firms worldwide can be categorised as a platform (Price Waterhouse Coopers, 2020).[1] It is estimated that within the next 10 years, 30% of gross economic output will stem from platforms such as Amazon, Alibaba and Facebook (Fong et al., 2020).

These platforms are important coordination devices, not only on a national level but also worldwide. Although the European Union has a suitable infrastructure and a large user base, companies from the US and Asia usually monetarise the market potentials (Price Waterhouse Coopers, 2020). Furthermore, in the digital economy there are rapid innovation cycles, which are known as "leapfrog competition" (Grave and Nyberg, 2017, 364). A firm with a dominant market position can be driven out of the market by a new company that offers goods and services more in line with the preferences of customers. Myspace vs. Facebook and Yahoo! vs. Google are examples. To include the near future of the rise of platform business models in the picture, a look at unicorn companies can be helpful (Büchel and Rusche, 2020a). Unicorn companies are not publicly listed but have a valuation of at least $1 billion based on recent funding rounds. The interest of investors can point to companies that can challenge dominant incumbent firms or enter new markets.

In the first quarter of 2020 there were 471 unicorns worldwide with a combined valuation of $715 billion. From those 471 unicorns, 135 (with a total valuation of $307 billion) could be classified as platforms.[2] Accordingly, the mean valuation of a unicorn is $1.52 billion, while a platform unicorn has a mean valuation of around $2.3 billion. With regard to regional distribution, only 29 of all 471 unicorns and 12 of the 135 platform unicorns come from the EU27. So, the dominance of Asian and US firms in the European digital economy is likely to continue.

## Gatekeepers and proposed obligations

The fast growth and the large economic power of digital platforms have led to a discussion on whether this can cause economic or societal problems that need to be tackled by new regulations (e.g. HM Treasury, 2019; United States House of Representatives, 2020). The recent proposals for an adopted regulation by the European Commission (2020a, 2020b) showed that the European Union answered the question on the need for new

1 Only the Saudi Arabian Oil Company (Saudi Aramco) and Berkshire Hathaway do not have a platform business model.

2 Büchel and Rusche (2020a) based on data from CB Insights.

regulations affirmatively. This is justified by the dominant position of companies from the USA and Asia in the European Union. The setting of fair rules in the EU can create a level playing field where incumbent companies can compete fairly with digital companies with market power. A level playing field can also set the scene for the rise of start-ups for example from the EU that can enhance competition in the digital economy. Furthermore, the regulation of dominant digital platforms can be justified because they can act as a gatekeeper between their different groups of users and, therefore, can potentially influence competition in their favour (Demary et al., 2020).

The Digital Markets Act (European Commission, 2020b) directly aims at core platform services of only a small number of digital platforms which are called gatekeepers. According to Article 3 of the proposal (European Commission, 2020b, 36), a platform is a "gatekeeper if

(a) it has a significant impact on the internal market;

(b) it operates a core platform service which serves as an important gateway for business users to reach end users; and

(c) it enjoys an entrenched and durable position in its operations or it is foreseeable that it will enjoy such a position in the near future."

In Article 2(2) of the proposal, eight services are defined that can be deemed a core platform service including examples such as online search engines, advertising services and video-sharing platform services (European Commission, 2020b, 35-36). Furthermore, to judge whether there is indeed a significant impact on the internal market, quantitative metrics or a qualitative case-by-case analysis can be used (European Commission, 2020b, 2). Once a platform is a gatekeeper under the definition of the proposal, the European Commission (2020b, Art. 3(7)) identifies the core platform services where problems are evident. For these services, Article 5 lists general obligations and Article 6 lists additional obligations, respectively, that can be specified further by the Commission. The general obligations state that

- The gatekeeper is not permitted to combine personal data of the core platform service with the data from other platform services or third-party sources without the consent of the user.

- It is mandatory to give business users the opportunity to offer their services on other intermediation services at other or better conditions.

- There exists the possibility for business users to promote offers and conclude contracts with customers acquired by the core platform service whether they use the core platform service for that purposes or not. Conversely, customers can access content, subscriptions etc. with the business user whether they acquired them via the core platform service or not.

- It is forbidden to prevent business users from raising issues with public authorities.

- It is forbidden to prescribe the use of identification services offered by the gatekeeper.

- The gatekeeper refrains from prescribing the use of any other service of the gatekeeper.

- The platform provides advertisers and publishers for whom it supplies advertising services, upon their request, with information concerning the price paid by the advertiser and publisher, as well as the amount or remuneration paid to the publisher.

Article 6 also stipulates that the gatekeeper will refrain from using data in competition with business users that is not publicly available. Additionally, it is allowed for end users to uninstall and pre-install software applications and software application stores as long this does not affect the essential functioning of the device or operation system. If the company fails to comply with these obligations laid down in Article 5 and 6, the Commission can, according to Article 26 of the proposal, impose a fine of up to 10% of global turnover of one year.

### General analysis of the proposal

Before the proposed regulations are discussed in detail, it is necessary to point to the fact that the proposed regulation applies once a gatekeeper has evolved. This, however, leaves out the fact that shortcomings in merger control were to some extent responsible for the rise of digital platforms. This is highlighted by 566 completed acquisitions by Apple, Amazon, Google and Facebook listed in a report of the United States House of Representatives (2020) and 400 acquisitions by Amazon, Apple, Facebook, Google and Microsoft within the last 10 years listed in a report for the British HM Treasury (2019). Both reports, however, have not found a prohibited acquisition. An acquisition of an incumbent firm is especially problematic in the digital economy. Since the value of data increases with its integration with data from different sources (Krotova et al., 2019), digital companies that already have a lot of data are willing to pay higher amounts for start-ups with valuable datasets. Furthermore, companies on a platform market must reach a critical mass of users first in order to be successful. In such a situation, the acquisition of a company with an established product that has almost reached this critical mass is problematic for competition. This is due to the fact that the acquiring platform does not have the pressure to innovate in order to improve its position.

Accordingly, a better use of the means of merger control can also help to keep competition working. This can also help to prevent the abuse of a dominant position by gatekeepers because it keeps the doors open for new competitors and helps to prevent a leveraging of market power in new markets. Keeping markets open with the help of merger control replaces to some extent the need to intervene in the future. Nevertheless, in the current situation where dominant platforms have evolved, there is indeed the need to intervene once the dominant position causes structural competition problems.

From an analytical viewpoint, there are structural problems if there are high entry or start-up costs, which include high switching costs for consumers coupled with a high level of concentration in the market.

High entry or start-up costs may be due to positive network effects (attracting a critical mass of consumers in order to be attractive for more consumers), regulatory barriers, lack of relevant data or other inputs and users that are hard to attract (for example, due to high switching costs or single-homing consumers). From an economist's perspective, high entry costs are no problem for competition if multiple enterprises are active in a market. The competition between these enterprises limits the market power of any incumbent firm directly. This is done by, for example, the introduction of standards that reduce switching costs, the setting of low prices or investments in innovation.

If the market is concentrated, there is no problem for competition if start-up or entry costs are low. In the case of monopolistic behaviour and monopoly profits, new competitors are attracted, and customers are also prone to switch to these new competitors due to high prices and the tendency for less innovation and lower quality in monopolistic markets. For example, although Alphabet (Google) dominates the market for advertisement-supported videos uploaded by private users with YouTube, TikTok was able to successfully enter this market with new features (Büchel and Rusche, 2020b).

Thus, high market concentration and high entry costs reinforce each other and can cause structural competition problems. In the platform economy, this can manifest itself in the emergence of increasingly vertically integrated platforms and the formation of proprietary markets. In a proprietary market, a platform is also a competitor to its own business users, and it shapes a business environment in its own favour to maximise its profits.

However, innovation and changes in demand can still lead to functioning competition. If a dominant firm does not constantly adapt to changing consumer interests or invest in new or improved existing products, it will create an opportunity for new competitors to enter the market. Accordingly, a case-by-case assessment is needed to take into account all features of the market and possible substitutes so as to judge whether there are indeed structural competition problems that have to be addressed by market interventions. This also means that interventions should be evidence-based and are not capable of limiting the growth opportunities of (potential) start-ups.

## Specific analysis of the proposal

Furthermore, since platforms are focal points and reduce transaction costs for the whole economy, the consequences of a regulation on the functioning of the whole platform ecosystem must also be considered. Against the backdrop of the Digital Markets Act, the definition of gatekeepers as such includes all important points from an analytical perspective. In another article, the authors have stipulated in a proposed gatekeeper definition that the platform's service must be of high relevance for the users (Büchel and Rusche, 2020a). The rationale behind including the relevance is that a company can only act as a gatekeeper and can potentially abuse their power if it keeps a gate the users want to pass or must pass. However, since this includes arbitrariness to some extent and the judgement is subjective, it is reasonable to concentrate on the important gateway proposed by the Commission.

Often, a platform acting as a gatekeeper captures a large share of total revenue in one of the underlying markets. It must be agreed upon by the authors that the Commission does not only look at the revenue that is generated in the consumer market by the platform when it decides upon whether the gatekeeper has a significant impact. A collection of alternative measures was set out by the recent Progress Report of the Expert Group for the Observatory on the Online Platform Economy (2021). This includes measures like the share of consumer attention, by quantifying the number of users or the amount of time users spend on the platform. Even the number of acquisitions undertaken by the platform as a competition strategy can serve as an adequate measure.

A platform acting as a gatekeeper can exert its gatekeeper role with regard to consumers just as well as to business users. This is due to the platform's large impact on the entire sector, which extends not only to the business-user side of the platform but also to consumers and to potential platform user groups such as advertisers, who should also be included in the analysis.

As a result of the above analysis, it is clear that platforms in the digital economy will gain in power and importance, so much so as to even deem platforms an essential facility. Note that the regulations in European competition law in Article 102 of the Treaty on the Functioning of the European Union (TFEU) already ascribe platforms the status of an essential facility and regulate them accordingly (Demary et al., 2019). This can include the implementation of fair rules, mandatory data sharing and a right to use the platform. Accordingly, most obligations laid down in the proposal can be based on this Article or other regulations already implemented (Grave and Nyberg, 2020). The question arises whether there is still value added by the added obligations in the proposal for a Digital Markets Act. And indeed, the value added by the proposal is that it eradicates the uncertainty of the rules to be applied in Single Market to some extent.

The proposal lays down the allowed conduct by powerful digital platforms and therefore also lays down the rights users have on the platform. The specific obligations, especially in Article 5, are based on cases and proceedings in European competition law. The internal separation of data was prescribed by the Bundeskartellamt in Germany in a case against Facebook and was also implemented in German laws (Bundesgesetzblatt, 2021, 2-32). The ability to apply better conditions is based on the cases regarding best price clauses in the EU and in member states, for example.[3] This, however, also means that the Digital Markets Act is not the endpoint in efforts to regulate the digital economy. Innovation and new products or services may also lead to new practices that may be deemed to be anti-competitive in the future.

## Conclusion and policy recommendations

The question of whether the current competitive framework is sufficient to address issues raised in digital markets is closely connected to the question of whether the authorities' abilities are sufficient to proceed effectively against platforms that abuse their market power and breach existing competition rules. Generally, the current regulatory framework is also capable of acting in the Digital Age and it builds on established legal pillars. Nevertheless, tailored modernisation and adaption, for example in merger control, are helpful to ensure fair competition. In addition, there is a need for tailored procedures on individual large online platforms with gatekeeper power, on a case-by-case basis, by using the existing regulatory instruments. The Digital Markets Act proposal also helps to regulate the digital economy with its powerful platforms. This is done by a pre-emptive definition of fair rules based on existing experience by competition authorities. This also makes faster interventions in digital markets possible. While this can be problematic because it can potentially limit the positive effects of platforms and curbs innovations in the digital economy, the fact that it is based on evidence and can be targeted to specific situations can help to create a competitive digital economy and therefore enhance innovations.

In addition, based on the analysis above, policy recommendations can be made that can be used to further improve competition. These recommendations can be divided into: (1) how to deal with dominant digital platforms in the market now; and (2) how to keep markets open and promote future innovation.

First, the COVID-19 pandemic and the measures taken by the governments to combat it have improved the market positions of many digital platforms. In the short run, their often dominant position and their market power cannot be limited by e.g. new competitors or merger control. But the following policy recommendations can be useful for adapting the proposed Digital Markets Act.

It is beneficial to have one or only a few platforms in a market rather than having many small platforms. This, however, makes setting and enforcing clear and distinct rules more important as attempted by the Digital Markets Act. Constantly changing regulations, the introduction of undefined legal concepts or different competing regulations that can potentially apply for one firm only foster *legal uncertainty* and limit growth opportunities. Therefore, there should be no legal uncertainty because it limits investments and makes agents avoid reasonable transactions.

*Pre-emptive state intervention in the economy must be avoided*. Governmental interventions in markets can ultimately result in markets being designed according to political aims, limiting competition and therefore harming consumers by reducing the availability of goods and services. This especially includes interventions without there being a dominant firm or (likely) infringements in the market. This increases uncertainty for economic agents, damages competition and limits incentives for investments and innovations. The obligations in the Digital Markets Act that are based on experiences by authorities and only aim at a limited number of big platforms are an efficient way to regulate and can be a blueprint for further regulations.

3 For example, Bundeskartellamt (2015), best price clause of online hotel portal Booking.com also violates competition law.

*Pay attention to the role of state-owned enterprises*. State-owned enterprises and enterprises with backup from their home country are also left out of the discussion of the proposed regulation. State-owned enterprises, including platforms, may be formed or supported by governments outside the European Union and can, among other things, raise challenges to competition in the EU. This factor has to be considered in the discussion on how to adapt competition rules in the Digital Single Market.

Second, in the long run, there are more means available in order to react to identified problems for competition on digital markets. The following steps can be useful.

*Avoid too tight and too many detailed regulations*. Generally, anti-trust regulation is a channel for new firms and, hence, innovation. The regulation keeps markets open and creates a level playing field where fair competition can take place. Anti-trust regulations prevent dominant firms from sealing their markets off from competition. Nevertheless, regulations that are too tight limit the opportunities and incentives for new firms and can, therefore, harm consumers by restricting innovation and the availability of products and services. Accordingly, before new rules or regulations are implemented, the effects of previously implemented rules and regulations should be considered and evaluated.

*Take merger control into consideration with new regulations or new competition tools*. Merger control can be empowered by including data and other synergies between involved enterprises and assessments. This could, as an extreme measure, include a moratorium on mergers and acquisition involving big digital players.

*Promote the Single Market in the EU*. One reason for the low number of competitive digital platforms from the EU is the incomplete Single Market. Fragmentation into many small markets limits growth opportunities and the scaling up of business models. Therefore, less money is invested in the EU, and investment is allocated to different projects in different member states. This fragmentation is exemplified by huge price differences for Pampers diapers on Amazon in different member states (Economist, 2020). Pampers diapers are a standardised product that can easily be shipped and that can be ordered on a digital platform from any country. In a completed single market, huge price differences should vanish because consumers in high price countries order in countries with lower prices. The fact that this is not the case shows that the Single Market in the EU is incomplete. Accordingly, promoting the Single Market supports competition by laying the groundwork for start-ups that challenge the position of dominant digital platforms.
